# A comprehensive evaluation model for the intelligent automobile cockpit comfort

**DOI:** 10.1038/s41598-022-19261-x

**Published:** 2022-09-02

**Authors:** Jianjun Yang, Shanshan Xing, Yimeng Chen, Ruizhi Qiu, Chunrong Hua, Dawei Dong

**Affiliations:** 1 School of Automobile and Transportation, Xihua University, Chengdu, 610039 China; 2 School of Mechanical Engineering, Southwest Jiaotong University, Chengdu, 610031 China; 3 Vehicle Measurement, Control and Safety Key Laboratory of Sichuan Province, Chengdu, 610039 China

**Keywords:** Engineering, Mathematics and computing

## Abstract

Under the background of automobile intelligence, cockpit comfort is receiving increasing attention, and intelligent cockpit comfort evaluation is especially important. To study the intelligent cockpit comfort evaluation model, this paper divides the intelligent cockpit comfort influencing factors into four factors and influencing indices: acoustic environment, optical environment, thermal environment, and human–computer interaction environment. The subjective and objective evaluation methods are used to obtain the subjective weights and objective weights of each index by the analytic hierarchy process and the improved entropy weight method, respectively. On this basis, the weights are combined by using the game theory viewpoint to obtain a comprehensive evaluation model of the intelligent automobile cockpit comfort. Then, the cloud algorithm was used to generate the rank comprehensive cloud model of each index for comparison. The research results found that among the four main factors affecting the intelligent automobile cockpit comfort, human–computer interaction has the greatest impact on it, followed by the thermal environment, acoustic environment, and optical environment. The results of the study can be used in intelligent cockpit design to make intelligent cockpits provide better services for people.

## Introduction

Automobiles play a very important role in people's daily lives. With the rapid development of artificial intelligence, internet technologies and communication technologies, intelligent automobiles will occupy an important position and be competitive in the automobile industry^1^. Intelligent technologies are rapidly evolving with the help of the latest information technologies, which are turning simple and ordinary automobiles into intelligent automobiles^2^. The development of intelligent automobiles aims to achieve "safe and comfortable next-generation automobiles" that have not only the common features of the automobiles themselves but also the functionality of intelligent technologies^3^. In the future, autonomous driving technology will change the traditional driving pattern, the driver will be freed from boring driving operations, and the automobile cockpit will become a place for rest, entertainment and work. The main trends in the development of intelligent automobiles are to ensure safety, comfort, efficiency and environmental sustainability^4^. Eventually, with the integration of information and communication technologies, intelligent automobiles will become standardized^5^.

People's demand for comfort in automobiles is increasing, and comfort has become an important factor influencing passengers' choice of intelligent automobiles^6,7^. For drivers, driving comfort is related not only to satisfaction with the driving experience but also to driving safety and the long-term health of the driver^8^. For passengers, passenger comfort is a major concern for road automobile passengers because it affects people's health and productivity^9^. In the current context, improving driving safety and comfort are very persistent themes with in-automobile design^10^. Passenger comfort analysis plays a pivotal role in the automobile evaluation process. Passenger comfort as a subjective perception is a complex term that includes factors such as thermal environment comfort and acoustic environment comfort^11–13^. Among them, the thermal environment has an impact on the efficiency and comfort of personal travel^14^. The acoustic environment has an impact on people performing complex behaviors; noise can cause stress and interfere with attention, and the acoustic environment also affects passengers' cognitive performance^15^.

Many factors affect passenger comfort. It has been found that the optical environment affects the physiological and behavioral patterns of occupants. As the primary environmental cue to the body's master biological clock, the optical environment affects sleep, mood, performance, alertness, quality of life, and health in different populations^16–18^. The factors influencing passenger comfort can be considered equally influential for intelligent automobile cockpit comfort^19^. Manning et al. in 2021 pointed out that the complexity of human–computer interaction is an important factor in evaluating the layout of intelligent cockpits^20^. For the detection of passenger comfort, most existing studies focus on physical factors affecting comfort, such as sitting position, vibration and noise, but few scholars have used physiological signals between humans and the driving environment to detect intelligent automobile cockpit comfort^21^. In indoor human comfort evaluation, the acoustic environment, optical environment, and thermal environment are considered the three main physical factors affecting human comfort^22^. In the evaluation of intelligent automobile cockpit comfort, the scientific and comprehensive selection of indicators has an important influence on the accuracy and objectivity of the evaluation results. Therefore, a multifactor comprehensive evaluation method should be used to comprehensively evaluate the intelligent automobile cockpit comfort from multiple aspects, such as acoustic environment, optical environment, thermal environment, and human–computer interaction.

As the automobile becomes increasingly intelligent, research on the evaluation model of intelligent automobile cockpit comfort is also very important. After the evaluation indices are selected, it is necessary to find a suitable evaluation method for each index weight. To date, the determination of index weights is roughly divided into subjective assignment methods and objective assignment methods^23,24^. The subjective assignment method is a method in which decision-makers directly give preference information, such as the analytic hierarchy process (AHP), least squares and the Delphi method^25–27^. The objective assignment method is a method based on the information of the decision matrix, such as the entropy weighting method, multi-objective optimization method, principal component analysis, and the multiple attribute decision-making model (MADM)^28–31^. Among them, AHP was used to evaluate the relative importance among various services provided by intelligent automobiles, such as driver assistance, infotainment, and IoT hubs^32^. As a subjective empowerment method, AHP is widely used in multicriteria decision-making and is suitable to help solve decision problems characterized by many interrelated factors^33,34^. However, the subjective weight determination method has the disadvantage of being influenced by the subjective preferences of decision-makers^35^. The entropy weight method was used to measure the complexity of human–computer interaction in intelligent cockpits and calculated the weight distribution of specific metrics that affect the complexity of human–computer interaction inside intelligent cockpits^20^. A multicriteria decision model, as an objective assignment method, was used to evaluate the feasibility and effectiveness of the cockpit ergonomic layout^36^. In the objective evaluation method, the entropy weight method mainly uses the size of the entropy value in information theory to represent the uncertainty of the message. It can calculate the ability of each evaluation attribute to transmit decision-making information, and calculate the relative weight between attributes^37–40^. In addition, the entropy method itself has inherent defects, namely: (1) when experts believe that a certain indicator has no impact on the intelligent automobile cockpit comfort, there will be a situation in which the traditional entropy method calculation equation is restricted; and (2) when experts believe that different indicators have the same degree of impact on the intelligent automobile cockpit comfort, there will be a situation in which the entropy value jumps, making the evaluation of the intelligent automobile cockpit comfort unrealistic. Due to these inherent defects, when using the entropy weight method to evaluate the intelligent automobile cockpit comfort, correction coefficients ${K}_{1}$ and ${K}_{2}$ should be introduced to improve the traditional entropy weight method.

The combination weights derived from the game theory-based combination assignment method can take into account the objective properties of the program indicators while fully considering the subjective tendencies of decision-makers. The result of minimizing the deviation of the subjective and objective weights is the result of a Nash equilibrium after a noncooperative game with subjective and objective weights^41^. To evaluate the intelligent automobile cockpit more comprehensively, AHP was used to calculate subjective weights, objective weights were calculated using the improved entropy weighting method. And game-theoretic combined weights were introduced to construct a comprehensive evaluation model for the intelligent automobile cockpit comfort.

## Materials and methods

### Evaluation index system construction

**Figure 1:**
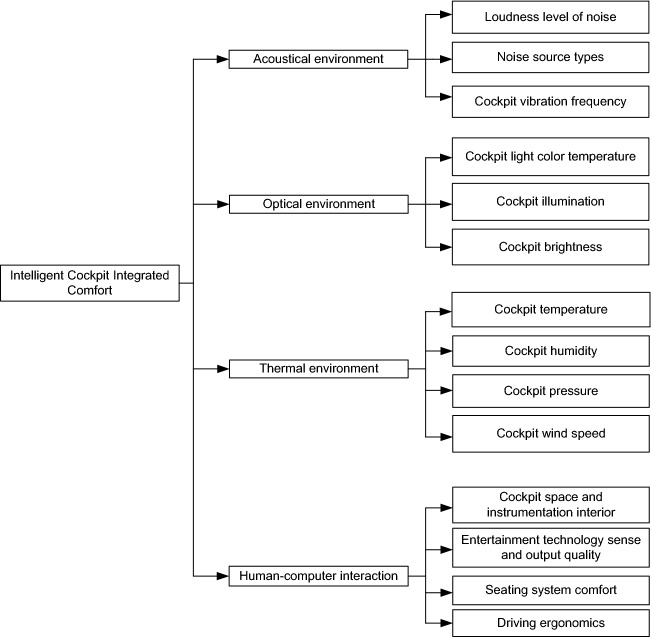
Comprehensive evaluation index system for impact of intelligent automobile cockpit comfort.

The intelligent automobile cockpit comfort impact evaluation system is complex. Too many or too few indicators are not conducive or do not facilitate the scientific, reasonable, and accurate judgment and evaluation of the system. When there are too few indicators, it is impossible to accurately reflect the judgmental attributes of the system. When there are too many indicators, it will make the judgment attributes of the system too complicated. With the indoor environmental comfort evaluation as a reference, a comprehensive evaluation system for the impact of intelligent automobile cockpit comfort is established according to the ASHRAE standard and the standard proposed by the international standards organization TC205 in 2005, as shown in Fig. 1^42,43^. The evaluation system includes four primary indicators, i.e., the acoustic environment, optical environment, thermal environment and human–computer interaction, and 14 secondary indicators.

### Subjective weights

The steps to calculate the weights using AHP methods are as follows. Step 1: Decompose complex problems into multielement problems. Step 2: Group these elements to create a hierarchical analysis model. Step 3: Construct a judgment matrix $A$, compare any two elements using the 1–9 scale method, and obtain the importance of each indicator at each level. Step 4: Calculate the maximum eigenvalue vector and subjective weights of the judgment matrix using mathematical methods. Step 5: Based on the consistency index $CI$ calculation, the consistency of the judgment matrix is tested, expressed as $CI=\left({\lambda }_{max}-n\right)/\left(n-1\right)$, and the average random consistency index $RI$. If the random consistency ratio $CR=CI/RI <0.10$, then the consistency of the judgment matrix is considered acceptable; otherwise, the judgment matrix should be appropriately revised.

### Objective weights

The objective weight is calculated by the entropy weight method, and the rating of the $j$th evaluation index among the $i$th experts is recorded as ${x}_{ij}$ when using entropy weight for evaluation. The steps of calculating the objective weights are as follows:

1. The weight of the $j$th evaluation index in the rating value of the $i$th expert is recorded as ${p}_{ij}$, which is calculated as:
  1 $${p}_{ij}=\frac{{x}_{ij}}{\sum_{i=1}^{n}{x}_{ij}}.$$

Since $0\le {p}_{ij}\le 1$, then $0\le -\sum_{i=1}^{n}{p}_{ij}\mathrm{ln}{p}_{ij}\le 1,0\le {H}_{j}\le 1$. If experts believe that a certain indicator has very little or even no effect on the intelligent automobile cockpit comfort, there will be a case when ${p}_{ij}=0$. In this case, $\mathrm{ln}{p}_{ij}$ is meaningless. Therefore, this paper introduces a correction coefficient ${K}_{1}$, which modifies the equation as follows:

2 $${p}_{ij}^{ {^{\prime}}}=\frac{\left({p}_{ij}+{K}_{1}\right)}{\sum_{i=1}^{n}\left({p}_{ij}+{K}_{1}\right)}.$$

Among the models that use the improved entropy weight method in a decision, there is a risk evaluation model based on the improved entropy weight and a decision model in terms of risk evaluation, both of which take the correction coefficient ${K}_{1}$ as ${10}^{-4}$ and achieve good evaluation results^44^. Therefore, to correct Eq. (1) in this paper, we take the value of ${K}_{1}$ as ${10}^{-4}$. The introduction of this correction factor ${K}_{1}$ avoids the extreme case where the expert believes that a certain indicator has no effect on the intelligent automobile cockpit comfort and makes $\mathrm{ln}{p}_{ij}$ meaningless. It solves the limitations of the traditional equation and has less impact on the entropy value ${H}_{j}$.

- Calculate the entropy value ${H}_{j}$ of the $j$th indicator:
  3 $${H}_{j}=\frac{1}{\mathit{ln}\left(n\right)}\sum_{j=1}^{m}{p}_{ij}^{ {^{\prime}}}ln{p}_{ij}^{ {^{\prime}}}.$$
- Calculate the objective weights of the indicators ${\omega }_{j}$:
  4 $${\omega }_{j}=\frac{1-{H}_{j}}{\sum_{j=1}^{m}\left(1-{H}_{j}\right)}.$$

This equation has an inherent deficiency. It occurs when experts believe that the degree of influence of each indicator on the intelligent automobile cockpit comfort is very similar, that is, when the weights of the four indicators of acoustic environment, optical environment, thermal environment and human–computer interaction are very close. At this time, when the indicator entropy value ${H}_{j}$ is close to 1($j=\mathrm{1,2},3\dots ,m)$, the small difference between ${H}_{j}$ and the others will cause the corresponding entropy weight of each to change exponentially, and the phenomenon of entropy jump occurs, making the intelligent automobile cockpit comfort level not match the actual situation. If a larger positive correction factor ${K}_{2}$ is added to the numerator and denominator at the same time, the above entropy jump phenomenon can be corrected. The entropy weight equation in this case will change to:

5 $${\omega }_{j}=\frac{1-{H}_{j}+{K}_{2}}{\sum_{j=1}^{m}(1-{H}_{j}+{K}_{2})}\left({K}_{2}>0\right).$$

${ K}_{2}$ must be considered when taking the value of the entropy change and cannot affect the final established intelligent automobile cockpit comfort evaluation model. In the research model of the importance of network assets based on objective empowerment, ${K}_{2}$ is taken as $\frac{1}{10}\sum_{j=1}^{m}\left(1-{H}_{j}\right)$ and achieves good results^45^. At the same time, it is calculated that it will not have a large impact on the original entropy value. Therefore, to correct Equation (5) in this paper, we take the value of ${K}_{2}$ as $\frac{1}{10}\sum_{j=1}^{m}\left(1-{H}_{j}\right)$.

### Combined weights

The subjective weights and objective weights of the intelligent automobile cockpit comfort evaluation index can be calculated by using AHP and improved entropy weight method, respectively. According to the idea of game theory, the subjective and objective weights are fused to seek the optimal solution of the weights and obtain the combined weights of the intelligent automobile cockpit comfort impact evaluation index. The specific steps are as follows. With $s$ methods to assign weights to each evaluation indicator, the $s$ assigning methods obtain the sum of the $s$ evaluation index weight vector:

6 $${W}_{k}=\left({W}_{k1},{W}_{k2}\dots {W}_{ki}\right)\left(k=\mathrm{1,2},3,\dots ,s\right).$$

Any linear combination of the sum of s vectors is given by:

7 $$W=\sum_{k=1}^{s}{\alpha }_{k}{W}_{k}^{T} \left(k=\mathrm{1,2},\dots ,s\right).$$

Solving for the optimal weight coefficient ${\alpha }_{k}$, for which the countermeasure model is introduced:

8 $$\mathrm{min} \left\| \sum_{k=1}^{n}{\alpha }_{k}{W}_{k}^{ T}-{W}_{l}^{ T}\right\| \left(l=\mathrm{1,2},\dots ,n\right)$$

Based on the properties of differentiation, the optimal first-order derivative condition for the above Equation (8) is derived as:

9 $$\sum_{k=1}^{n}{\alpha }_{k}{W}_{l}{W}_{k}^{T}={W}_{l}{W}_{K}^{T}\left(l=\mathrm{1,2},\dots ,n\right).$$

According to Eq. (9), $\left({\alpha }_{1}, {\alpha }_{2}, \dots, {\alpha }_{n}\right)$ can be calculated and then normalized to obtain the weighting coefficients:${\alpha }_{k}^{,}={\alpha }_{k}$/${\sum }_{k=1}^{n}{\alpha }_{k}$. Then, the game-theoretic-based combination weight ${W}^{^{\prime}}=\sum_{k=1}^{n}{\alpha }_{k}^{,}{W}_{k}^{T},k=\mathrm{1,2},3\dots ,n$. The final obtained intelligent automobile cockpit comfort evaluation model ${W}^{^{\prime}}={\alpha }_{1}^{,}{W}_{1}^{T}+{\alpha }_{2}^{,}{W}_{2}^{T}$, where ${\alpha }_{1}^{,}$ is the optimal weight coefficient of the AHP method and ${\alpha }_{2}^{,}$ is the optimal weight coefficient of the improved entropy weight method. ${W}_{1}$ is the subjective weight derived using the AHP method, and ${W}_{2}$ is the objective weight derived using the improved entropy weight method.

### Principle of cloud model

Li De-Yi et al. proposed a cloud model based on the cross-pollination of probability theory and fuzzy geometry theory. The cloud model uses the three numerical feature values of cloud expectation ${E}_{x}$, entropy ${E}_{n}$ and super entropy ${H}_{e}$ to react to the uncertainty and vagueness of passenger comfort evaluation, and the transformation of qualitative concepts and quantitative expressions is carried out through the cloud generator^46^. Expectation ${E}_{x}$ is the most typical sample point in the passenger comfort evaluation domain, i.e., the mean value of the cloud drops; entropy ${E}_{n}$ is the measure of vagueness in passenger comfort evaluation, and the larger its value, the more vaguely the concept is expressed; and super entropy ${H}_{e}$ is the uncertainty measure of entropy ${E}_{n}$, i.e., the entropy of entropy, and the larger its value is, the greater the randomness of affiliation, responding to the association of vagueness and randomness.

## Intelligent automobile cockpit comfort evaluation example study

### Expert evaluation

**Figure 2:**
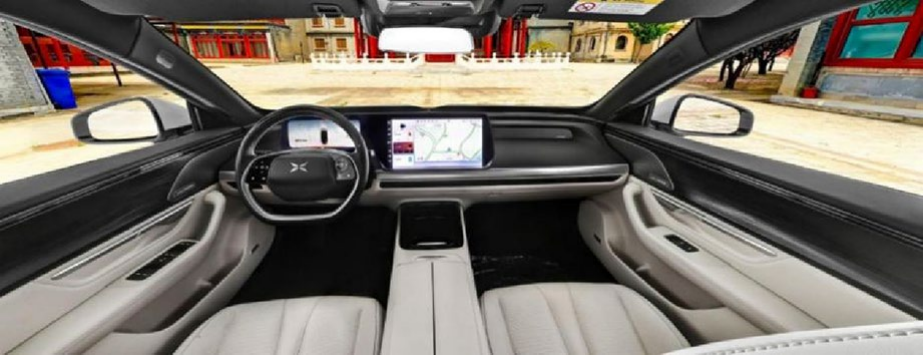
Interior view of the cockpit of the Xiao-Peng P7 automobile.

As a powerful intelligent automobile, the Xiao-Peng P7 has the four highlights of self-driving, human–machine interaction, the Internet of Everything and intelligent ecology and is considered the new standard for the second generation of intelligent automobiles in China; it is shown in Fig. 2. Five experts in the automobile field were invited to evaluate the intelligent cockpit comfort of the Xiao-Peng P7 based on different indicators reflecting their usual work and automobile riding experience. Based on a 10-point scale, and the higher the score, the greater the impact, the scoring results are shown in Table 1.

**Table 1:** Level 1 index score value.

	Acoustic environment	Optical environment	Thermal environment	Human–computer interaction
Expert 1	8.1	8.2	8.4	8.5
Expert 2	7.5	7.3	7.6	7.9
Expert 3	8.3	8.2	8.4	9
Expert 4	8.2	7.9	8	8.7
Expert 5	8.4	8.1	8.2	8.7

### Determination of subjective weights

The questionnaire was used to investigate the importance of each indicator in the criterion layer, and then the judgment matrix A was obtained:

$$A=\left[\begin{array}{cc}\begin{array}{cc}1& 1.19\\ 0.84& 1\end{array}& \begin{array}{cc}0.89& 0.78\\ 0.53& 0.60\end{array}\\ \begin{array}{cc}1.12& 1.88\\ 1.27& 1.66\end{array}& \begin{array}{cc}1& 0.81\\ 1.24& 1\end{array}\end{array}\right]$$

After calculating the maximum eigenvalue of the judgment matrix A: ${\uplambda }_{\mathrm{max}}=4.0159$, the consistency index CI = 0.0053. Table 2 shows the average random consistency index $RI=0.89$, the consistency ratio $CR=0.0059<0.10$, and the consistency ratio of the judgment matrix is acceptable. The subjective weight is ${\upomega }_{1}=\left(\mathrm{0.2337,0.1762,0.2792,0.3109}\right)$.

**Table 2:** Standard evaluation level numerical characteristics.

Comprehensive evaluation level	Intolerable	Very uncomfortable	Uncomfortable	Slightly uncomfortable	Comfortable
Interval	$[\mathrm{0,2})$	$[\mathrm{2,4})$	$[\mathrm{4,6})$	$[\mathrm{6,8})$	$\left[\mathrm{8,10}\right]$
${E}_{x}$	1	3	5	7	9
${E}_{n}$	0.33	0.33	0.33	0.33	0.33
${H}_{e}$	0.05	0.05	0.05	0.05	0.05

### Determination of objective weights

By the improved entropy weight method, the objective weight is calculated by substituting the score value of the first-level index into Equations (1)–(5), which is recorded as ${\omega }_{2}$, by calculating the objective weight: ${\omega }_{2}=\left(\mathrm{0.2383,0.2707,0.2192,0.2719}\right)$.

### Determination of combination weights

Using the idea of combining weights from game theory, the index weights obtained from AHP and the entropy weight method are combined to obtain the optimal weight coefficients ${\alpha }_{1}=0.7944$ and ${\alpha }_{2}=0.2168$. These coefficients are then normalized, leading to ${\alpha }_{1}^{,}=0.7872$ and ${\alpha }_{2}^{,}=0.2128$. Using the equation ${W}^{^{\prime}}={\alpha }_{1}^{,}{W}_{1}^{T}+{\alpha }_{2}^{,}{W}_{2}^{T}$, the final combined weight ${W}^{^{\prime}}$= (0.2347,0.1963,0.2664,0.3026) is obtained.

### Calculating the similarity

**Figure 3:**
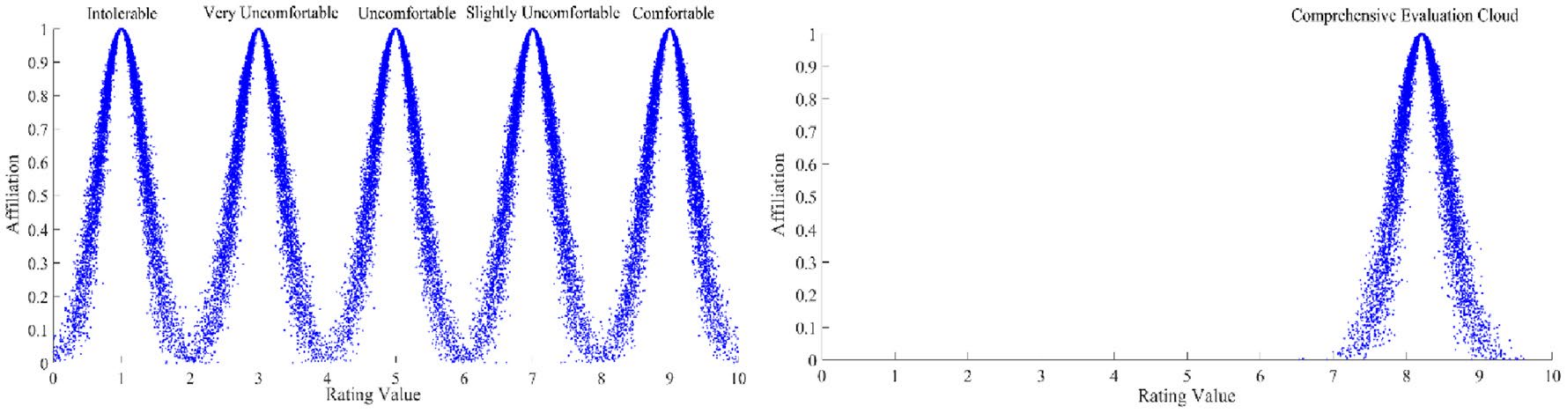
(**a**) Standard evaluation cloud; (**b**) Comprehensive evaluation cloud.

Subjective scales have been used for a long time for comfort evaluation^47–50^. According to relevant standards and subjective evaluation scale specifications related to human engineering, combined with the actual situation of the automobile cockpit, the standard indoor environment comfort division, reference to the existing reference literature and consultation with experts, the evaluation criteria for the evaluation of the intelligent automobile cockpit comfort indicators are formulated^51,52^. The criteria then are divided into "intolerable", "very uncomfortable", "uncomfortable", "slightly uncomfortable" and "comfortable", and the corresponding evaluation intervals are [0,2), [2,4), [4,6), [6,8), and [8, 10]; from unbearable to comfortable, the intelligent automobile cockpit comfort is improving. From equation ${E}_{{x}_{i}}=\frac{{d}_{{min}_{i}}+{d}_{{max}_{i}}}{2}\mathrm{and }{E}_{{n}_{i}}=\frac{{d}_{{max}_{i}}-{d}_{{min}_{i}}}{6},$ the intelligent automobile cockpit comfort evaluation standard cloud digital features can be obtained, as shown in Table 2; the standard evaluation cloud map is generated by the MATLAB forward cloud generator, as shown in Fig. 3a. Through the forward cloud generator and inverse cloud generator proposed by Chinese academician Li De-Yi in 1995^46^, the comprehensive evaluation cloud map of the intelligent automobile cockpit comfort and the evaluation cloud of each index can be generated; the comprehensive evaluation cloud of the intelligent automobile cockpit comfort is shown in Fig. 3b.

The similarity between the comprehensive evaluation cloud and the standard cloud is calculated separately, and the evaluation level corresponding to the standard cloud with the greatest similarity is the final evaluation result. The similarity between the comprehensive evaluation cloud and the standard evaluation cloud was calculated as shown in Table 3.

**Table 3:** Similarity between integrated evaluation cloud and standard evaluation cloud.

Standard evaluation cloud	Intolerable	Very uncomfortable	Uncomfortable	Slightly uncomfortable	Comfortable
Similarity	0.000	0.000	0.000	0.0024	0.0757

**Figure 4:**
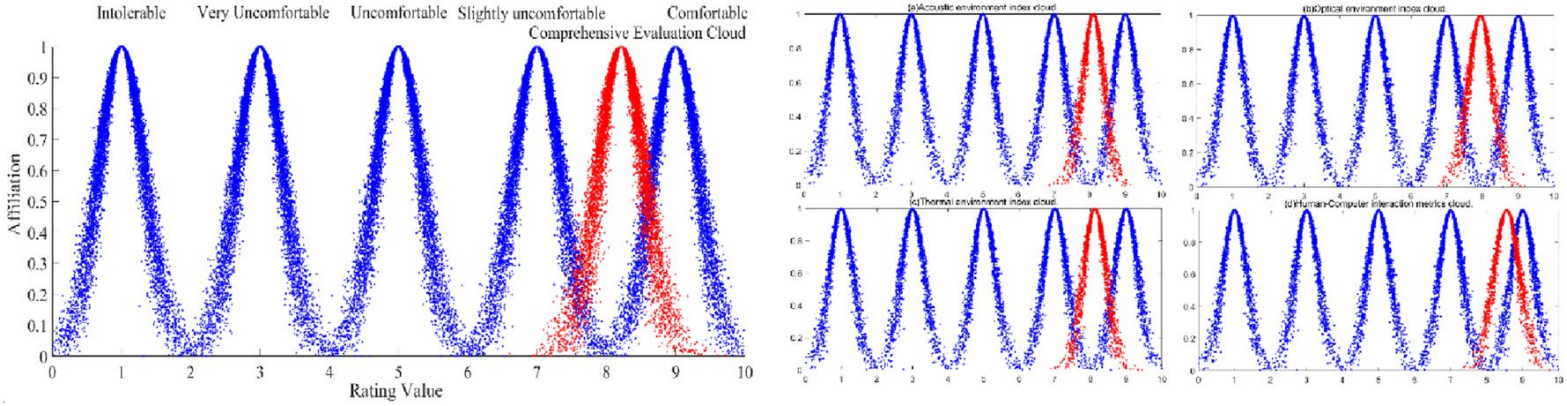
(**a**) Comparison of each indicator evaluation cloud with standard evaluation cloud. (**b**) Comparison between each indicator evaluation cloud and standard evaluation cloud.

Table 3 shows that the degree of comfort identified by the comprehensive evaluation cloud and the standard evaluation cloud rated as comfortable have the highest similarity, so the final evaluation result can be considered comfortable, but there is still some room for improvement. Meanwhile, through the MATLAB forward cloud generator, the cloud diagram was drawn by using the comprehensive evaluation cloud digital features and each standard evaluation cloud digital feature, and a similar comparison diagram was obtained, as shown in Fig. 4a. The cloud diagram can be more intuitively seen as consistent with the similarity calculation result. By drawing the similarity comparison diagram between each level of indicators and each standard cloud, it can be more intuitive to see the similarity between each level of indicators and each evaluation standard level for acoustic environment, optical environment, thermal environment and human–computer interaction, as shown in Fig. 4b.

## Results and discussion

In this paper, AHP is firstly used to determine the subjective weight of the comfort evaluation index of the intelligent automobile cockpit comfort. In the process of using the entropy weighting method to calculate the target weight, an improved algorithm is proposed. Compared with the traditional algorithm, it ensures that $\mathrm{ln}{P}_{ij}$ is mathematically meaningful, and controls the influence of the entropy value within a reasonable range, reducing artificial factors, and consider the actual situation of the evaluation indicators. Finally, the idea of game theory is introduced to determine this combination of weights. This approach fully considers that the subjective experience and objective inherent properties of the evaluation indices make the index weight replication more reasonable, which is conducive to the comprehensive evaluation of the intelligent automobile cockpit comfort. Among the four primary indicators affecting the intelligent automobile cockpit comfort, the human–computer interaction design has the greatest influence on the intelligent cockpit, followed by the thermal environment design, acoustic environment design and optical environment design.

In this paper, we use the intelligent automobile cockpit comfort evaluation model to evaluate the cockpit comfort of the Xiao-Peng P7 model automobile, and the four indicators are evaluated as comfortable in a comprehensive manner, as shown in Fig. 4a. From Table 4, we know that among the four first-level indicators, the acoustic environment, thermal environment and human–computer interaction environment can be considered comfortable, and only the optical environment design is considered slightly uncomfortable. This indicates that the intelligent cockpit design of the Xiao-Peng P7 intelligent automobile needs to focus on the influence of its optical environment aspects.

**Table 4:** Similarity between each index evaluation cloud and standard evaluation cloud.

	Intolerable	Very uncomfortable	Uncomfortable	Slightly uncomfortable	Comfortable
Acoustic environment similarity	0.000	0.000	0.000	0.0026	0.0301
Optical environment similarity	0.000	0.000	0.000	0.0302	0.0094
Thermal environment similarity	0.000	0.000	0.000	0.0040	0.0378
Human–computer interaction similarity	0.000	0.000	0.000	0.0002	0.4819

The intelligent cockpit comfort model based on game theory combination weights designed in this paper improves the intelligent automobile cockpit comfort impact evaluation system, which has certain reference value for future automobile engineers to design and manufacture intelligent cockpit. However, this paper has the following shortcomings in the work of evaluating the intelligent automotive cockpit comfort:

1. When calculating the objective weights of the acoustic environment, optical environment, thermal environment, and human–computer interaction using the improved entropy weight method in this paper, only five experts were invited to conduct the comfort level. The number of experts is small and has certain limitations. In the future, when further research work on the intelligent automotive cockpit comfort is carried out, the number of experts needs to be increased to make the evaluation of the intelligent automotive cockpit comfort more reasonable.
2. Due to the reason of the length, this paper only considers the influence of the primary indicators such as acoustic environment, optical environment, thermal environment, and human–computer interaction on the intelligent automotive cockpit comfort. In future research work, the automotive intelligent cockpit comfort will be further studied from the secondary indicators.

## Data Availability

All data generated or analyzed during this study are included in this published article.
